# Local Magnetic Hyperthermia and Systemic Gemcitabine/Paclitaxel Chemotherapy Triggers Neo-Angiogenesis in Orthotopic Pancreatic Tumors without Involvement of Auto/Paracrine Tumor Cell VEGF Signaling and Hypoxia

**DOI:** 10.3390/cancers16010033

**Published:** 2023-12-20

**Authors:** Wisdom O. Maduabuchi, Felista L. Tansi, Bernd Faenger, Paul Southern, Quentin A. Pankhurst, Frank Steiniger, Martin Westermann, Ingrid Hilger

**Affiliations:** 1 Department of Experimental Radiology, Institute of Diagnostic and Interventional Radiology, Jena University Hospital, Friedrich Schiller University Jena, Am Klinikum 1, 07747 Jena, Germanyfelista.tansi@med.uni-jena.de (F.L.T.); bernd.faenger@med.uni-jena.de (B.F.); 2 Resonant Circuits Limited, 21 Albemarle Street, London W1S 4BS, UK; psouthern@resonantcircuits.com (P.S.); q.pankhurst@ucl.ac.uk (Q.A.P.); 3 Healthcare Biomagnetics Laboratory, University College London, 21 Albemarle St., London W1S 4BS, UK; 4 Center for Electron Microscopy, Jena University Hospital, Friedrich Schiller University Jena, Ziegelmuehlenweg 1, 07743 Jena, Germany; frank.steiniger@med.uni-jena.de (F.S.); martin.westermann@med.uni-jena.de (M.W.)

**Keywords:** pancreatic adenocarcinoma (PDAC), angiogenesis, magnetic hyperthermia, chemotherapy, orthotopic tumor model, hypoxia

## Abstract

**Simple Summary:**

The treatment of pancreatic tumors is challenging because they are poorly vascularized and because this inhibits drug delivery. Using mild magnetic hyperthermia in combination with chemotherapy, we show that drug accessibility can be increased by the transient induction of new blood vessel growth in the tumors. By examining molecular and physiological factors in an orthotopic tumor model in mice, we show that the combination therapy results in local cellular stress, and that blood vessel growth is triggered by cell-to-cell communications within the treated tumors. This mechanism is quite different from the usually reported driver of tumor vessel formation—*viz.* hypoxia—and may therefore offer a means of modulating both the extent and the location of the tumor vasculature for therapeutic benefit. We anticipate that combined chemo- and thermo-therapy may in the future improve the efficacy and tolerability of pancreatic cancer therapies.

**Abstract:**

There is a growing interest in exploring the therapeutically mediated modulation of tumor vascularization of pancreatic cancer, which is known for its poorly perfused tumor microenvironment limiting the delivery of therapeutic agents to the tumor site. Here, we assessed how magnetic hyperthermia in combination with chemotherapy selectively affects growth, the vascular compartment of tumors, and the presence of tumor cells expressing key regulators of angiogenesis. To that purpose, a orthotopic PANC-1 (fluorescent human pancreatic adenocarcinoma) mouse tumor model (Rj:Athym-Foxn1nu/nu) was used. Magnetic hyperthermia was applied alone or in combination with systemic chemotherapy (gemcitabine 50 mg per kg body weight, nab-pacitaxel 30 mg/kg body weight) on days 1 and 7 following magnetic nanoparticle application (dose: 1 mg per 100 mm^3^ of tumor). We used ultrasound imaging, immunohistochemistry, multi-spectral optoacoustic tomography (MSOT), and hematology to assess the biological parameters mentioned above. We found that magnetic hyperthermia in combination with gemcitabine/paclitaxel chemotherapy was able to impact tumor growth (decreased volumes and Ki67 expression) and to trigger neo-angiogenesis (increased small vessel diameter) as a result of the therapeutically mediated cell damages/stress in tumors. The applied stressors activated specific pro-angiogenic mechanisms, which differed from those seen in hypoxic conditions involving HIF-1α, since (a) treated tumors showed a significant decrease of cells expressing VEGF, CD31, HIF-1α, and neuropilin-1; and (b) the relative tumor blood volume and oxygen level remained unchanged. Neo-angiogenesis seems to be the result of the activation of cell stress pathways, like MAPK pathways (high number of pERK-expressing tumor cells). In the long term, the combination of magnetic hyperthermia and chemotherapy could potentially be applied to transiently modulate tumor angiogenesis and to improve drug accessibility during oncologic therapies of pancreatic cancer.

## 1. Introduction

Pancreatic cancer remains a lethal disease, with as many deaths as new cases [1]. Over 80% of pancreatic cancers are classified as pancreatic ductal adenocarcinoma (PDAC), and the majority of patients are diagnosed with metastatic or unresectable tumors [1,2] of poor prognosis [3,4]. PDACs are very resistant to oncological treatments.

Pancreatic cancers have typically a tumor microenvironment (TME) that exhibits acute desmoplastic responses, reduced vascularization, altered immune cell infiltration, and intricate connections between stroma cells and extracellular matrix proteins [5,6]. This severe desmoplasia prevents adequate vascularization [7]. PDAC vasculature further presents collapsing lymphatic vessels and capillaries as a result of high interstitial pressure, with this paucity of adequate vasculature contributing to tumor hypoxia [8].

Nowadays, tumor vascularization is an important focus of interest when exploring therapeutically mediated modulations to improve tumor drug delivery, enhance the treatment efficacy, and overcome the inherent resistance of these tumors to various therapies. Blood vessels in tumors originate from pre-existing ones (angiogenesis) and this process is critical for the growth, invasion, and metastasis of pancreatic adenocarcinoma [9,10]. In this view, there is some tumor-angiogenic activity in PDAC when compared to chronic pancreatitis or normal tissues [11,12,13]. Therefore, anti-angiogenic tumor therapies have been established by inhibiting the formation of new blood vessels or blocking the activity of pro-angiogenic factors. Beyond this, disruption of tumor vascularity can also be used to improve tumor drug penetration and efficacy. Some examples are the combination of magnetic hyperthermia and chemotherapy in a variety of cancers (including colorectal cancer [14], breast cancer [15], oral carcinoma [16], glioblastoma [17], and pancreatic cancer [18]) in preclinical studies.

In general, intratumoral hypoxic regions seem to be one of the main known drivers of the tumor’s own angiogenesis [19]. This includes the upregulation of hypoxia-inducible factor-1α (HIF-1α) [20,21], which in turn upregulates the expression of angiogenic factors such as vascular endothelial growth factor (VEGF) [22], platelet-derived growth factor (PDGF), and fibroblast growth factor (FGF), among others [21]. In PDAC, the hypoxic microenvironment is the result of the substantial desmoplastic reactions and the severe extracellular matrix (ECM), which constricts blood vessels [2,10]. Despite this, there is restricted knowledge on how tumor angiogenesis can be more specifically modulated by defined anti-tumor treatments.

Interestingly, the tumor vasculature was reported to be a good target for anti-tumor hyperthermia. Examples are the induction of vascular stasis and dilatation [23,24], increased blood flow, and improved cellular uptake of chemotherapeutics [25,26]. Hyperthermic therapies are based on the therapeutic raising of the temperature (locally or regionally) for curative objectives [26,27]. Among them, magnetic hyperthermia (MH) is a particular type of thermal therapy in which magnetic nanoparticles (MNPs) are delivered to a tumor and subsequently subjected to an alternating magnetic field (AMF) to generate heat via specific magnetization processes [26,28,29]. Magnetic hyperthermia enables a highly localized heating of cancerous tissues [29,30].

Delivering magnetic hyperthermia in PDAC needs a highly localized intratumoral MNP deposition, as PDAC sites are near vital organs, and the relatively strong heating effects should be confined only to the tumor. To ensure sufficient tumor heating, many challenges and potential risk factors should be considered. These include the following: the MNP concentration; the retention of the MNPs over time; the extent of the heating volume; the MNP heating parameters; and the amplitude and frequency of the applied magnetic field [29,30]. Moreover, we previously showed that by adopting pulsatile thermal dosing (using a pulsed high–low sequence for the activation of the MNPs by the applied magnetic field), it is possible to significantly reduce the potentially damaging flow of heat away from the tumor [30]. Nevertheless, there is limited knowledge on how magnetic hyperthermia in combination with systemic chemotherapy is able to modulate the angiogenesis of pancreatic cancer.

Moreover, one of the most interesting developments is the discovery that autocrine and paracrine VEGF signaling occurs in tumor cells and that this signaling contributes to key aspects of tumorigenesis, especially the function of cancer stem cells, independently of angiogenesis. Signaling downstream of VEGF in tumor cells is mediated by VEGF receptor tyrosine kinases and neuropilins. This means that the function of VEGF seems not to be limited to angiogenesis but to be associated with the modulation of the immune response to favor growth and escape from immune surveillance [31].

In view of the highly dense and poorly perfused tumor microenvironment of PDAC, in this study we assessed how local tumor magnetic hyperthermia in combination with systemic administration of gemcitabine plus nab-paclitaxel (termed as “bimodal therapy”) selectively affects several biological parameters, namely (a) tumor cell growth (via tumor volume and proliferation activity of surviving tumor/stromal cells), (b) the vascular compartment of tumors (via new vessel formation, relative tumor blood volume, tumor oxygen supply, etc.), and (c) the auto/paracrine activity of surviving tumor/stromal cells (by assessing the expression of key regulators for tumor angiogenesis: VEGF, VEGFR2, HIF-1a, and pERK). We further analyzed how each of the single therapy modalities (chemotherapy or magnetic hyperthermia) contributed to the mentioned parameters.

## 2. Materials and Methods

### 2.1. Magnetic Nanoparticles

We used sterile multicore dextran-coated magnetic nanoparticles (MNPs, RCL-01, Resonant Circuits Limited, London, UK). The MNP parameters according to the supplier were as follows: (a) the iron content was 72 mg_Fe_/mL_fluid_, (b) the hydrodynamic diameter was 149 nm, (c) the polydispersity index (PDI) was 0.26, and (d) the intrinsic loss power (ILP) was 5.1 nHm^2^/kg_Fe_ as measured in an AMF of amplitude 5 kA/m at 1.0 MHz.

### 2.2. Orthotopic Pancreatic Tumor Model in Mice

All experiments were approved by the regional animal care committee (Thüringer Landesamt für Verbraucherschutz, Bad Langensalza, Germany, registration number UKJ-17/030) and executed in conformity with the international guidelines on the ethical use of animals. Animals received food and water ad libitum and were kept at room temperature (25 °C) under artificial day and night cycles (14 h light/10 h dark cycles). The induction of the orthotopic pancreatic tumor model was as follows: first, fluorescent PANC-1 cells (2 × 10^6^, ATCC, Manassas, VA, USA) in 2% (*v*/*v*) Matrigel™ (Corning Life Sciences, Corning, NY, USA) in PBS were surgically injected into the pancreas of 8- and 10-week-old female nude mice (Rj:Athym-Foxn1nu/nu, Janvier, Germany). Tumor localization and weekly assessment of tumor volume were confirmed by ultrasound imaging (Vevo700, FUJIFILM Visual sonics Inc., Toronto, ON, Canada). Animal body weight (BW) was measured weekly as a control for animal welfare.

### 2.3. Animal Groups and Experimentation Procedure

We used five independent experimental groups containing randomly selected animals: (1) MH-sC: mice received magnetic hyperthermia (MH) following intratumoral application of MNPs (RCL-01, 1 mg Fe per 100 mm³ tumor volume) and weekly systemic chemotherapy (50 mg gemcitabine, i.p., 30 mg nab-paclitaxel, i.v., per kg body weight) for 7 weeks; (2) MH: mice treated with magnetic hyperthermia only; (3) sC: mice treated for 7 weeks with systemic chemotherapy only; (4) M: mice injected with MNPs only; (5) N: non-treated controls (no MNPs, MH, or sC). There were 5 mice per group (Table 1).

**Figure 1:**
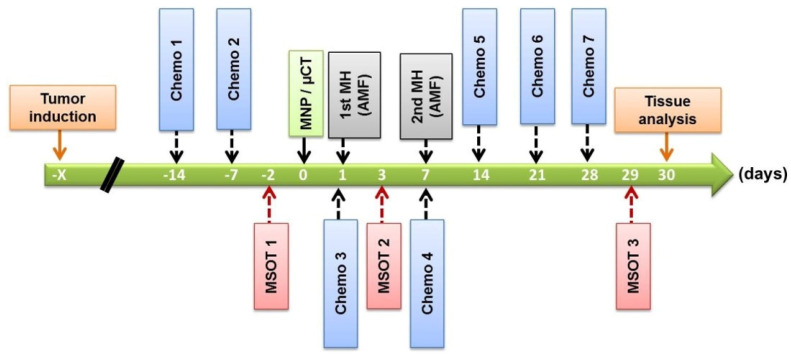
Timeline for animal experiments with the orthotopic PANC-1 tumor model. Chemo: systemic chemotherapy; MH: magnetic hyperthermia; MNP/µCT: magnetic nanoparticle injection followed by NIRF and CT imaging of animals. MSOT: multispectral optoacoustic tomography.

After orthotopic implantation into the pancreas, tumor volume measurements were conducted via ultrasound imaging. All tumor volume measurements were normalized to the respective tumor volume at experimental day 0 (Figure 1). Whole-body near-infrared optical and CT imaging (IVIS^®^ Spectrum CT, PerkinElmer, Inc., Hopkinton, MA, USA) was performed on day 0. The tumor treatment parameters are shown in Table 2. Finally, animals were sacrificed on the post-observation day 30. During all experimental procedures, animals were anesthetized with isoflurane (2–2.5 (*v*/*v*)%, CP-Pharma, Burgdorf, Germany).

### 2.4. Magnetic Hyperthermia and Calculation of Tumor Thermal Doses

After intratumoral injection of MNPs on day 0, magnetic hyperthermia treatments were carried out on day 1 and on day 7 (Figure 1). Time-varying magnetic fields *H*_o_ sin(2*π f t*) of amplitudes *H*_o_ = 5.43 and 8.34 kA/m were delivered using a solenoidal magnetic field generator (Preclinical MACH System, Resonant Circuits Limited, London, UK) operating at *f* = 1.048 ± 0.010 MHz. Fiber optic probe thermometers (TS5 and FOTEMPMK-19, Optocon AG, Dresden, Germany) were used to monitor and record the temperature readings. The highest temperature for each magnetic hyperthermia cycle was recorded using a temperature sensor probe that was pressed against the mouse skin (not piercing the skin) at an ultrasound-determined distance of 1.0 ± 0.1 mm from the tumor edge. To improve the localization of the thermal dose, the pulse hyperthermia method was employed as described by Tansi et al. [30]. Each hour-long hyperthermia sequence consisted of an initial step-wise increase of the magnetic field from zero to *H*_o1_ = 5.43 kA/m for 4 min, and then to *H*_o2_ = 8.34 kA/m for 11 min, followed by 14 cycles of (*H*_o1_ for  1 min and *H*_o2_ for 2 min), and finally 3 min at *H*_o1_, before returning to zero [30].

### 2.5. Transmission Electron Microscopy (TEM)

The excised tumors were cut into small pieces (diameter: 1 mm). Next, the tumor tissue samples were fixed with 4% (*v*/*v*) formaldehyde (Carl Roth, Karlsruhe, Germany) and 2.5% (*v*/*v*) glutaraldehyde solved in sodium cacodylate (0.1 M and pH 7.2, Serva, Heidelberg, Germany) for 3 h at room temperature and then placed at 4 °C for overnight incubation. Next, tissues were washed with sodium cacodylate buffer (3 times for 10 min each) and further fixed for 2 h at 20 °C with 1% (*w*/*v*) osmium tetroxide (Electron Microscopy Sciences, Hatfield, PA, USA). Next, an ascending ethanol series was employed to dehydrate tissue sections, and this was followed by staining the tissues with 2% (*w*/*v*) uranyl acetate diluted in 50% (*v*/*v*) ethanol. According to the manufacturer’s instruction, tissues were embedded in Araldite^®^ resin (Plano, Wetzlar, Germany). Afterwards, very thin sections were cut (70 nm in thickness) and mounted on carbon-coated 100 mesh grids (Qantifoil Mico Tools, Grossloebichau, Germany). The tissue sections were stained for 10 min with lead citrate and investigated at 80 kV in an electron microscope, Zeiss EM 902A (Carl Zeiss AG, Oberkochen, Germany).

### 2.6. Immunohistochemistry of Angiogenic Proteins Expressed in PANC-1 Tumors

To assess the impact of the therapy on the auto/paracrine VEGF signaling in pancreatic tumor cells, immunohistochemistry analysis of the expression of key protein players was performed. Since the PANC-1 cells were of human origin, antibodies against human epitopes of VEGF, VEGFR2, and neuropilin-1 were used. Animals were sacrificed at the end of therapy (day 30), and tumors were extracted, fixed, and embedded in paraffin. The embedded tumor tissue was then sliced into 3 µm sections (Microtome system, Microm HM 340E, Thermo-Fischer, Waltham, MA, USA) and placed on poly-l-lysin-coated glass slides. Tissue slices were dewaxed and subjected to antigen retrieval and blocking of endogenous biotin with avidin and biotin (Labeled Streptavidin–Biotin (LSAB)) or with endogenous peroxidase (DAB–3,3’-diaminobenzidine), depending on the colorimetric method used (see below). These were incubated with primary antibodies against human VEGFR2 (1:800, Cell Signaling Technology Inc., Danvers, MA, USA), mice CD31 (PECAM-1, 1:200, Cell Signaling Technology Inc., MA, USA), human/mouse HIF-1α (1:100, Cell Signaling Technology Inc., MA, USA), human Neuropilin-1, human α_v_β_3_ integrin (1:200, Abcam plc, Cambridge, UK), human VEGF (1:100, Invitrogen, Thermo Fisher Scientific, Waltham, MA, USA), or Ki67 (1:500, Abcam plc, Cambridge, UK), followed by incubation with a biotin-labeled secondary antibody (goat anti-rabbit IgG (H + L), 1:2250, Dianova GmbH, Hamburg, Germany) or with a peroxidase-labeled polymer secondary antibody (DAKO, Glostrup, Denmark). For the Labeled Streptavidin–Biotin colorimetric detection, Streptavidin Alkaline Phosphatase (Biozol, Freising, Germany) and Chromogen (DAKO, Glostrup, Denmark) were used for visualization, while for DAB (3,3′-Diaminobenzidine), a Chromogen (DAKO, Glostrup, Denmark) was directly used for antigen detection. The cell nuclei were counter-stained with hematoxylin (Sigma-Aldrich Chemie GmbH, Steinheim, Germany). Using a wide-angle dual Speed 2K CCD camera (TRS, Moorenweis, Germany), digitalized images were obtained. Semi-quantitative analysis of 8 micrographs per animal group and 2 animals per group was performed using Image J version 1.53k (NIH, Bethesda, MD, USA). The number of marker-positive cells was computed relative to the nuclei number (n = 973–1678 per image/ROI). Vessel diameter distribution was assessed by measuring the largest possible diameter of luminal space of cross-cut vessels in a ROI (area between 9.2–14.3 mm^2^).

### 2.7. Hemograms

At the end of therapy (day 30), blood was collected from the aorta of deeply isoflurane-sedated (of 5 % (*v*/*v*) mice using a 50 µL anti-coagulant Na-heparin capillary (Hirschmann Laborgeräte GmbH & Co. KG, Eberstadt, Germany). Blood was quickly transferred to a 200 µL isotonic salt solution (Fresenius Kabi AG, Bad Homburg, Germany) and measured immediately on an automated hematology analyzer for animals (Sysmex XT-1800i, Hyogo, Japan).

### 2.8. Multispectral Optoacoustic Tomography (MSOT)

MSOT was carried out to assess the level of oxygen within the tumor and the blood volume in the tumor tissue. The principle of optoacoustic imaging is based on the thermoelastic response of a sample to the absorption of pulsed laser energy. The resulting pressure wave can be detected by ultrasound transducers [32]. By detecting the thermoelastic response at several wavelengths, we can quantify the concentrations of oxygenated and deoxygenated hemoglobin molecules in red blood cells of the tumor tissue [33]. In the present study, a commercially available MultiSpectral Optoacoustic Tomography system for small animals was used for imaging (inVision 256-TF System, iThera Medical, Munich, Germany). The animal was placed in a right lateral recumbent position in the animal holder, which comprised a water impermeable transparent polyethylene membrane. The following wavelengths were used to stimulate the tissue and to measure the hemoglobin content in selected organs: 715, 730, 760, and 800 nm. MSOT was performed 3 times throughout the course of the experiments: at day −2 (2 days before MNP application (day 0)), day 2 (2 days after MNP application and 1 day after MH) and day 28 (end of therapy). In all cases, data on thermoelastic expansion were acquired at the position of the tumor. The images were reconstructed using a model-based algorithm integrated in the software (ViewMSOT™ v3.8, iThera Medical, Munich, Germany). Regions of interest (ROIs, size: 24.9 ± 1.5 mm^2^) at the tumor area were drawn, and linear spectral unmixing was performed using the multispectral processing option of the mentioned software. The resulting images were the result of a linear spectral unmixing algorithm, which determines the contribution of HbO_2_ and Hb to the signal in each pixel. The oxygen saturation (SO_2_; ratio expressed as fraction of 1) was calculated as follows:

$${SO}_{2}=\frac{C_{{HbO}_{2}}}{{(C}_{{HbO}_{2}}+C_{Hb})}$$

where *C_HbO_2__* and *C_Hb_* are the *HbO*_2_ and *Hb* signals (both in MSOT arbitrary units) averaged over all the pixels in the ROI; *C_HbO_2__* + *C_Hb_* reflects the total hemoglobin estimation (*Hb*_Total_). The ratio shown in the formula was used to increase the stability of the results [32]. Moreover, *HbO*_2_ and *Hb* signals of the tumor region were used to estimate semi-quantitatively the relative blood volume of the tumor (*C_HbO_2__* + *C_Hb_* = RBV) [34].

### 2.9. Statistics

Data were considered to be normally distributed and were represented as mean ± standard deviation or standard error of the mean. To determine the statistical significance between experimental groups, 1-way ANOVA and 2-way ANOVA tests were used, when normality and equal variance were applicable via the GraphPad 9 Prism software. Differences between groups were considered statistically significant when *p* < 0.05. The Pearson correlation coefficient (*r*) was used to measure the linear correlation between two variables, where values between 0 and 1 = positive correlation, 0 = no correlation, and 0 to −1 = negative correlation [35].

## 3. Results

### 3.1. Bimodal Therapy of Magnetic Hyperthermia and Chemotherapy Slowed Tumor Growth and Decreased Tumor Volume

**Figure 2:**
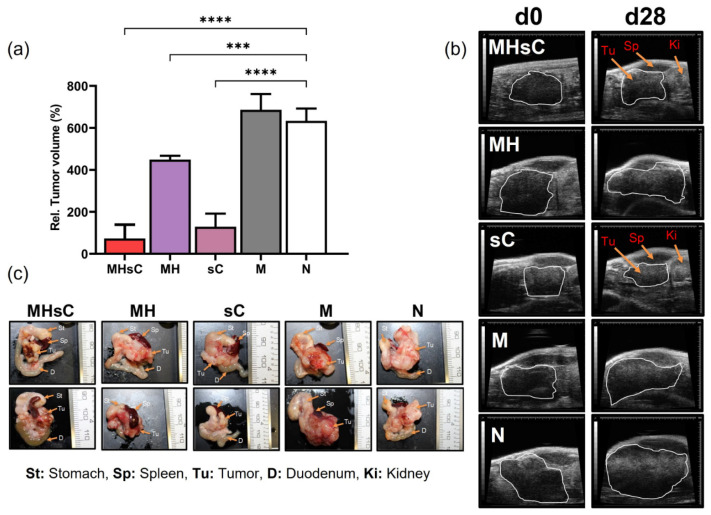
Both bimodal therapy and chemotherapy significantly inhibited pancreatic tumor growth. (**a**) Mean of relative tumor volumes determined over time from ultrasound images for the various animal groups are presented as percentages in relation to the tumor volume on day 0 before hyperthermia (MH1) therapy. *** *p* < 0.001; **** *p* < 0.0001 for day 28 in hyperthermia alone, MNP, and non-treated animal group compared to day 0. *n* = 5 per group. Each bar represents mean ± SD. (**b**) Representative ultrasound images of the tumors in each group. (**c**) Representative light images of excised tumors between the spleen and stomach, demonstrating a decrease in tumor volume after bimodal therapy and chemotherapy (caliper 1 mm). Tu: tumor; Ki: kidney. Animal groups: MHsC = magnetic hyperthermia + chemotherapy, MH = magnetic hyperthermia, sC = systemic chemotherapy, M = MNP alone, and N = non-treated.

In the mouse model of the orthotopic PANC-1 tumor, the bimodal therapy showed higher potential for decreasing tumor size and growth when compared to untreated animals (*p* < 0.00001, Figure 2a,b). Extracted tumors (day 30) were found to be positioned between the spleen, stomach, and kidney, as seen in the ultra-scan images (Figure 2c). All organs surrounding the pancreas were found to be morphologically intact at the time of excision.

Characteristic heating curve data from the two hyperthermia treatments applied to each animal (on days 1 and 7) are listed in Table 2, while Supplementary Figure S1 shows a representative temperature–time curve from these experiments, along with data on the anatomical localization of tumors (whole-body NIRF and CT imaging). The variation in tumor sizes (and therefore of the injected volumes of the MNPs) between the different animals and the fact that the temperature probe was positioned on the skin, ca. 1.0 mm from the edge of the tumors, makes direct comparison between the animal groups difficult. However, by plotting the observed amplitude of the temperature oscillation during the pulsed part of the heating sequence, ΔT_osc_, as a function of the injected volume of MNPs (a proxy for the tumor volume), it is clear that all of the experiments fall on the same trend line (see Supplementary Figure S2) and, furthermore, that the observed trend is comparable to that predicted by earlier theoretical modelling [30]. This indicates that the hyperthermia treatments as applied to both the MHsC and MH groups of animals were appropriate and commensurate with the initial tumor volumes of the individual animals.

**Figure 3:**
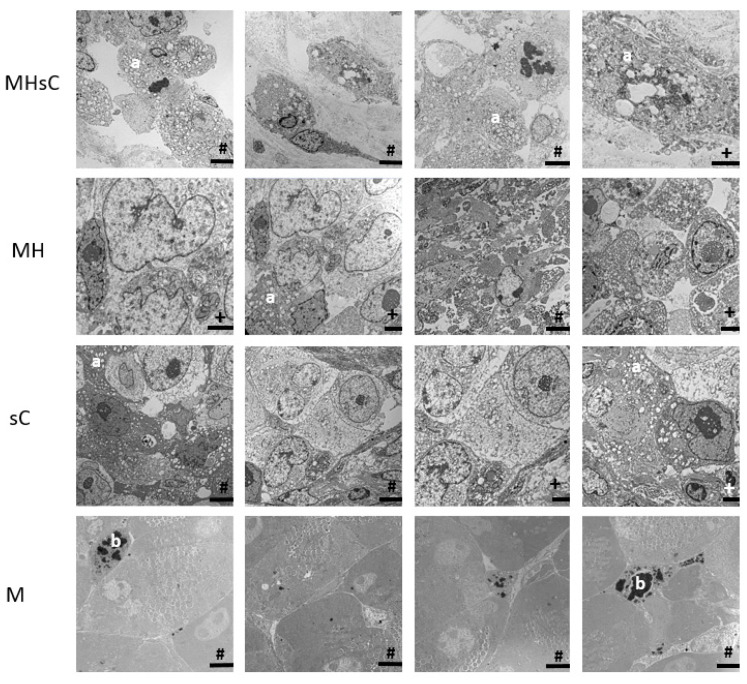
The different therapies induced varying degrees of cellular damage in the residual pancreatic tumor tissue. Tumors treated with MNPs only show intact cell-to cell connections and MNPs. (a) Cytoplasmatic vacuolization, (b) single cells (macrophages) with incorporated iron oxide nanoparticles. Animal groups: MHsC = magnetic hyperthermia + chemotherapy, MH = magnetic hyperthermia, sC = systemic chemotherapy, and M = MNPs alone. Scale: #: 5.0 µm, +: 2.5 µm.

The tumor therapy induced a distinct impact on the morphological constitution of tumor cells. In particular, transmission electron microscopy examination revealed cells with altered morphology of the cytoplasm (cytoplasmatic vacuolization and condensation, fragmentation of mitochondria, fragmentation of ER, etc.) in residual tumor tissues of all treated animal groups (bimodal therapy, magnetic hyperthermia, and chemotherapy) but more or less intact cell nuclei (no distinct chromatin condensation and margination). In contrast, tumors injected with the MNPs without treatment did not show signs of distinct cellular damage. Additionally, single cells (macrophages) were present with a high degree of incorporated MNPs, visible as intracellular MNP agglomerates (Figure 3).

### 3.2. Bimodal Therapy Reduces Intratumoral Angiogenesis in PANC-1 Orthotopic Tumors

**Figure 4:**
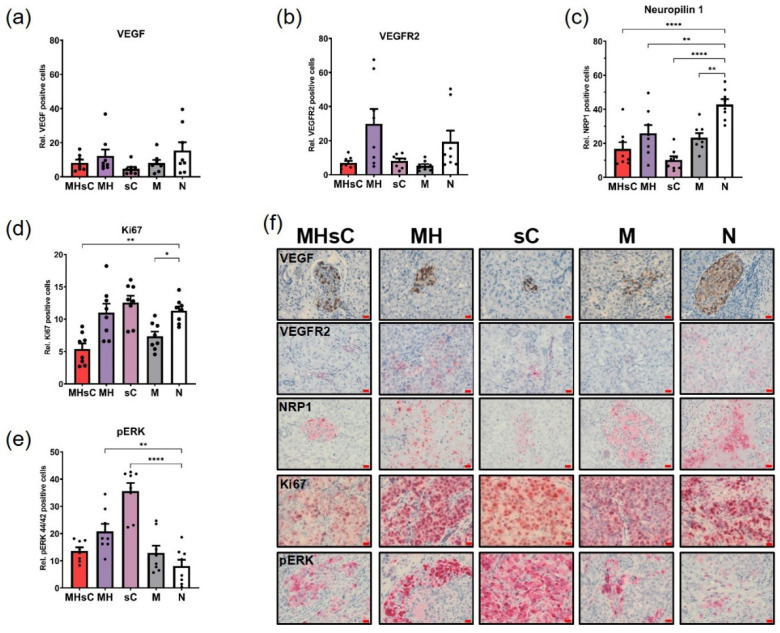
Magnetic hyperthermia combined with chemotherapy decreased intratumoral angiogenesis in residual pancreatic tumors. Semi-quantitative evaluation of intratumoral markers: (**a**) VEGF, (**b**) VEGFR2, (**c**) Neuropilin 1, (**d**) Ki67, and (**e**) phosphorylated ERK (pERK). Each bar represents mean ± SEM. Eight images were quantified per treatment group (*n* = 2 mice/group). Rel. = relative: Positively stained cells computed relative to the nuclei number in a ROI stained with hematoxylin using the Image-J software. (**f**) Representative histological micrographs of tumor tissue slices, scale bar: 20 µm. Animal groups: MHsC = magnetic hyperthermia + chemotherapy, MH = magnetic hyperthermia, sC = systemic chemotherapy, M = MNP alone, and N = non-treated. * *p* < 0.05, ** *p* < 0.01, **** *p* < 0.0001.

In general, the bimodal therapy (MHsC) reduced the expression of several important angiogenic markers. In particular, the tumor’s intracellular VEGF production was not stimulated by the bimodal therapy. There was no discernible increase or decrease in any group’s VEGF expressions, which were comparable across all of the animal groups (Figure 4a). Similarly, the bimodal therapy did not increase VEGFR2, which had low expression in the pancreatic tumor (Figure 4b). Tumors of the bimodal therapy, chemotherapy, and the MNP-alone animal group all showed a tendency to reduce VEGFR2 expression in the treated mice compared to the magnetic hyperthermia group and untreated animals (Figure 4b). Furthermore, expression of the co-receptor for VEGFR2, neuropilin-1 (NRP1), in tumor cells was considerably decreased in tumors after the bimodal therapy and chemotherapy alone. Accordingly, a 2.2- and 4-fold decrease (*p* < 0.0001) was seen, respectively, in comparison to the untreated tumors (Figure 4c). Animals given the bimodal therapy had a noticeably decreased level of the proliferation marker Ki67 compared to the non-treated animals (Figure 4d). Chemotherapy and magnetic hyperthermia, each as a single therapy modality, stimulated the ERK pathway in tumor cells. This was particularly pronounced in tumors of mice that received chemotherapy only (sC, *p* < 0.0001; Figure 4e). When compared to the untreated group, the expression of phosphorylated ERK was 3.8-fold higher in tumors of the chemotherapy-alone group and 2.4-fold higher in the magnetic hyperthermia-alone group (*p* < 0.01, Figure 4e). On the other hand, the bimodal treatment did not stimulate pERK. Consequently, the bimodal therapy of magnetic hyperthermia and chemotherapy was more effective than the individual therapies in suppressing tumor-derived angiogenesis.

### 3.3. Bimodal Therapy Decreased the Integrity of the Tumor Endothelium as Measured by the Intensity of CD31 Expression in the Endothelium

**Figure 5:**
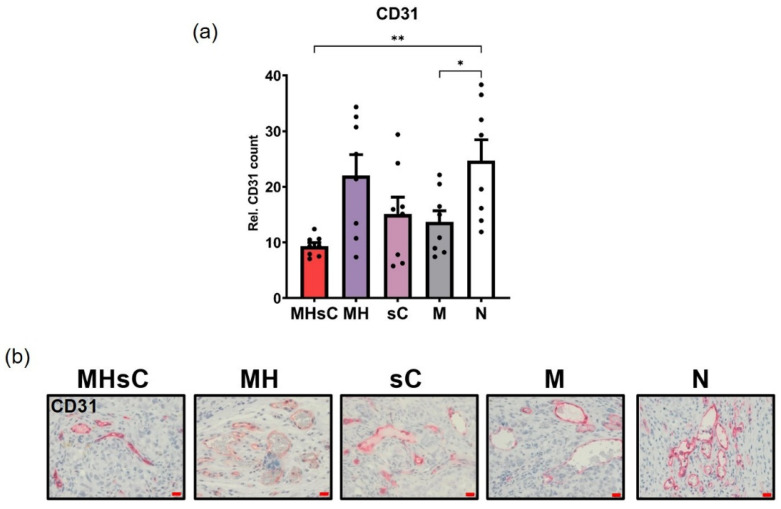
Chemotherapy in combination with magnetic hyperthermia significantly decreased the number of endothelial cells in residual pancreatic tumors. (**a**) Semi-quantitative analysis of the tumor’s CD31 expression levels. (**b**) Representative micrographs showing the number of CD31-positive cells in tumor tissue slices of the various treatment groups. Each bar represents mean ± SEM. Eight images were quantified per treatment group (*n* = 2 mice/group for CD31 expression analysis). Positive stains were computed relative to the nuclei number in a ROI stained with hematoxylin. Scale bar: 20 µm. Animal groups: MHsC = magnetic hyperthermia + chemotherapy, MH = magnetic hyperthermia, sC = systemic chemotherapy, M = MNP alone, and N = non-treated. * *p* < 0.05, ** *p* < 0.01.

Since the protein CD31 plays a role in the integrity of the endothelium [36], corresponding histological analysis of the expression intensity was performed. This revealed a substantial decrease in cells expressing CD31 following the bimodal therapy of magnetic hyperthermia and chemotherapy as compared to the CD31 level in tumors from the untreated animals. The mean number of CD31-positive cells of tumors in mice receiving bimodal therapy was 2.8 times lower (*p* < 0.01) than that of untreated animals (Figure 5a). Compared to the untreated control, chemotherapy or MNP-only animal groups showed a decrease in the number of CD31-positive cells in their tumors, whereas magnetic hyperthermia as single therapy modality had no effect in this aspect (Figure 5).

### 3.4. Bimodal Therapy Showed a Shift of Tumor Vessel Diameters towards an Increased Number of Smaller Vessels

**Figure 6:**
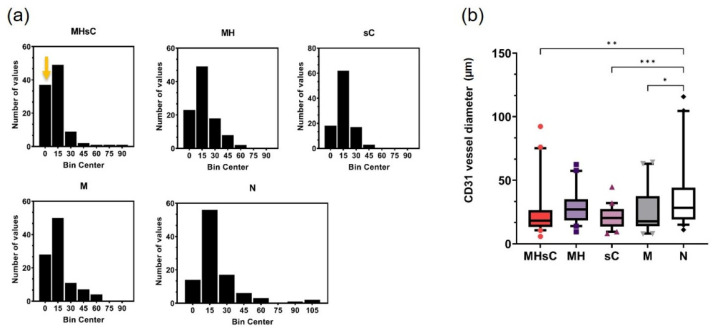
Bimodal therapy leads to a greater number of smaller blood vessels in residual pancreatic tumors than other treatments. (**a**) Histogram showing distribution of tumor blood vessels with regard to their diameters. The bimodal therapy group revealed a higher number of smaller tumor vessels of 0–5 µm diameter (area of analyzed ROI: 9.3 mm²) (MHsC, yellow arrow) as compared to those from the other treatment groups. (**b**) Semi-quantitative evaluation of CD31-positive tumor vessel diameter (0–120 µm). A bin is a bar whose height reflects the number of data points it contains; a bin center is the center value of the grouped data. Each box-plot represents mean ± SEM (*n* = 100 vessels/group). Animal groups: MHsC = magnetic hyperthermia + chemotherapy, MH = magnetic hyperthermia, sC = systemic chemotherapy, M = MNP alone, and N = non-treated. * *p* < 0.05, ** *p* < 0.01, *** *p* < 0.001.

Despite the loss in vascularity caused by the combination therapy of magnetic hyperthermia and chemotherapy (MHsC, Figure 5), diameter measurements of CD31-positive vessels show that residual tumors of the bimodal treatment group had a greater number of smaller vessels (*p* < 0.01) than those of the untreated animal group (Figure 6). This also applies for the animal group that received chemotherapy alone (*p* < 0.001) or MNPs alone (*p* < 0.05) as compared to the untreated control (Figure 6a). A closer examination of the tumor vessel distribution reveals that the bimodal group (yellow arrow) had the highest concentration of vessels with diameters between 0 and 5 µm when compared to the other groups (Figure 6b). The MNP-only control group and the magnetic hyperthermia-alone group followed, indicating a possible impact of nanoparticles. The majority of tumor vessel diameters ranged from 10 to 15 µm. However, the chemotherapy animal group was the only one lacking of tumor vessels larger than 60 µm (diameters, Figure 6a).

### 3.5. Bimodal Therapy Downregulates HIF-1α Expression and Hypoxic Regions within the Tumor

**Figure 7:**
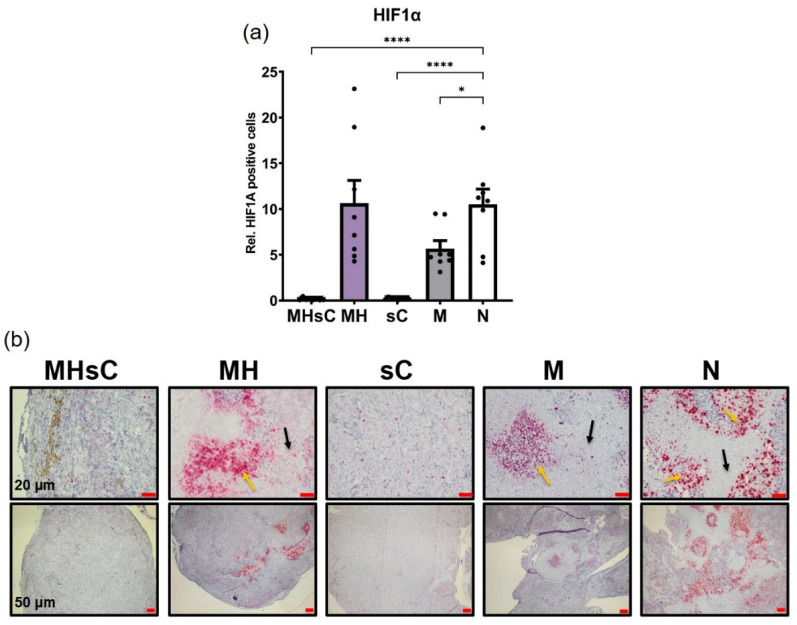
Bimodal therapy effectively abolished HIF-1α expression and hypoxic regions (necrotic areas with high HIF-1α concentrations) in residual pancreatic tumors. (**a**) Semi-quantitative evaluation of HIF-1α expression levels within the tumor slices computed in relation to the number of cell nuclei in a ROI (area: 9.3 mm^2^). Each bar represents mean ± SEM. Eight images were quantified per treatment group (*n* = 2 mice/group). Positive stains were computed relative to the cell (nuclei) number. (**b**) Representative histological micrographs of tumor tissue slices showing hypoxic regions. Animal groups: MHsC = magnetic hyperthermia + chemotherapy, MH = magnetic hyperthermia, sC = systemic chemotherapy, M = MNP alone, and N = non-treated. * *p* < 0.05, **** *p* < 0.0001. Yellow arrows = HIF-1α expression; black arrows: necrotic regions.

Considering that induction of tumor angiogenesis is known to be strongly related to the presence of hypoxia, we analyzed the expression of HIF-1α in treated and non-treated tumors. Interestingly, bimodal therapy completely abolished hypoxia, as seen in the number of HIF-1α-positive hypoxic tumor cells compared to those in non-treated tumors (*p* < 0.0001, Figure 7). This was comparable to HIF-1α levels in tumors from mice treated with chemotherapy alone (*p* < 0.0001). The exposure of tumors to MNPs also reduced the number of HIF-1α-positive cells (*p* < 0.05) as compared to non-treated controls. Contrarily, magnetic hyperthermia had no effect on hypoxia within the tumor, resulting in comparatively high levels of HIF-1α-expressing cells. In all cases, cells with HIF-1α were immunohistologically shown to be grouped in specific areas of necrotic cells, so-called “hypoxic regions”, except for the chemotherapy alone and the bimodal therapy animal group, wherein only very few hypoxic cells were spotted all over the tumor (Figure 7b). Necrotic cells were identified by a morphological appearance distinct from viable cells. Overall, the bimodal therapy abolished intratumoral hypoxia.

### 3.6. Tumor Blood Volume and Tumor Oxygen Saturation

Blood analysis revealed comparable levels of red blood cells and hemoglobin in the body of all animal groups. Both the bimodal therapy and hyperthermia alone had no appreciable impact on the number of red blood cells or hemoglobin in comparison to the untreated animals (Supplementary Figure S3). Equally, MSOT measurements revealed that the estimated oxygen saturation [34] in the tumor remained unchanged in all animal groups throughout the course of treatment (Supplementary Figure S4). There was correlation (Pearson correlation) between tumor oxygen saturation and the relative blood volume, (r = 0.883, *p* = 0.047) in the magnetic hyperthermia group (Supplementary Figure S5).

## 4. Discussion

The bimodal therapy magnetic hyperthermia and chemotherapy showed a unique therapeutic impact on pancreatic tumors, which was related to the biological parameters “tumor volume”, “cell proliferation”, “new vessel formation”, “expression of pERK”, and the “presence of HIF-1α-positive-cells” in the tumors. Conceiving the mentioned parameters as components of a so-called “therapeutic signature”, the therapeutic signature of the bimodal tumor therapy was mostly different in comparison to the single therapy modalities of chemotherapy and magnetic hyperthermia, and even the presence of MNPs in the tumor per se had some impact on the tumor tissue.

In particular, the reduction of tumor volumes after treatment with the bimodal therapy (MHsC animal group) is represented by the loss of cells due to irreversible damage. The remaining tumor mass was composed of severely damaged but living cells: electron micrographs showed signs of cellular apoptosis and autophagy (e.g., cells showing vacuolized cytoplasm [37]) and cells of reduced proliferation activity (reduced number of Ki67-positive tumor cells). Since the administered temperature doses were rather mild, the impact on tumor volume was particularly attributed to the systemic chemotherapy (50 mg/kg gemcitabine, i.p., and 30 mg/kg nab-paclitaxel, i.v.), as there was a largely reduced tumor volume after treatment with the bimodal therapy or chemotherapy alone.

At the same time, the bimodal therapy distinctly decreased the expression of CD31 in pancreatic tumors, which implicates a weakened integrity of the endothelium after exposure to the bimodal therapy. In particular, CD31 expression is involved in maintaining cell adhesion and junctions, regulating leukocyte trafficking, participating in mechano-transduction, and influencing angiogenesis in tumor endothelial cells [36]. At the same time, the bimodal therapy triggered the formation of new vessels (increased number of smaller vessels with diameters between 0 and 5µm). This effect was not HIF1α-dependent, as evidenced by the observation of a low HIF-1α expression in tumor tissue sections in the absence of necrotic areas with high HIF-1α concentrations, which is reportedly typical of PDAC tumors [2]. Interestingly, it was accompanied with, at least in tendency, an increased pERK expression. pERK is related to angiogenesis through several molecular pathways, including the TGF-1/pERK and VEGF/mTOR/Akt pathways, and/or to autophagy or cellular stress responses [38].

Therefore, we postulate that the bimodal therapy induced neo-angiogenesis-triggered glycogen consumption in surviving (e.g., autophagic) tumor cells through an HIF-1α-independent mechanism (absence of hypoxia), which was particularly driven by magnetic hyperthermia (see below). In this context, autophagy and glycolysis are both crucial metabolic processes, which are interconnected in several ways. For example, autophagy can be triggered to provide additional ATP through the hydrolysis of stored macromolecules when glycolysis is inhibited. Additionally, autophagy can also be involved in removing damaged mitochondria, which could subsequently affect glycolytic metabolism [39]. Interestingly, some studies have suggested that glycolytic intermediates such as lactate can stimulate angiogenesis in an adenosine-monophosphate-activated protein kinase (AMPK)-dependent mechanism [40]. This means that the severe cell damage induced in the tumor region via the bimodal therapy triggered tumor neo-angiogenesis as result of a cell stress response, via the mitogen-activated protein kinase (MAPK) pathway and not via the presence of HIF-1 α-positive tumor cells as usually seen in hypoxic conditions. Autocrine and paracrine VEGF signaling seems not to play a key role in this aspect (see below).

Magnetic hyperthermia alone (as in the MH animal group) also had an impact on tumor growth in the orthotopic model. This effect is related to mild hyperthermic temperature doses deposited in the tumors and is in agreement with a previous study on the treatment of orthotopic PANC-1 tumors with magnetic hyperthermia [30]. Since orthotopic pancreatic tumors are surrounded by vital organs in contrast to subcutaneous ones [41], the local hyperthermic temperatures must be carefully controlled, and the deposition of mild temperatures seems to be favorable to fulfill this goal. Moreover, the pancreas is surrounded by highly vascular organs responsible for a distinct thermal convection during therapy [42]. In this context, by employing pulsatile thermal dosing, it is possible to distinctly reduce the potentially damaging flow of heat away from the tumor [30]. Interestingly, the mild temperature doses applied to tumors had almost no effect on cell proliferation (Ki67, in comparison to non-treated controls), whereas higher temperature doses can have this effect (see, e.g., [24]). Consequently, the comparatively mild hyperthermic conditions stressed tumor cells rather than destroying them. It appears likely, therefore, that the presence of viable (proliferating, Ki67-positive) tumor cells drove the formation of new vessels (small diameters between 0–5 µm). There is some evidence that stressed stromal tumor cells release pro-inflammatory cytokines and growth factors that promote angiogenesis [43,44]. It is very likely that such relationships also apply for PDAC [45]. These pro-angiogenic effects were accompanied by a distinct increase of pERK in tumor cells, presumably as result of cellular stress in the absence of hypoxia (unchanged HIF-1α levels, SO_2_, relative blood volume, VEGF, and VEGFR2 expression in tumor cells, compared to untreated controls; see below). In agreement with this, a good correlation was seen between the SO_2_ value with increasing relative blood volume, which is related to the capacity of hyperthermia to dilate tumor vasculature [5] and which increases the blood supply to tumors, allowing for greater oxygenation and therapeutic access [46]. In summary, magnetic hyperthermia may have been the major contributor in inducing a tumor cell stress-mediated neo-angiogenesis in the orthotopic pancreatic tumors.

Regarding chemotherapy as a single therapeutic modality (as in the sC animal group), the distinct reduction of tumor volume can be associated with the action of gemcitabine leading to tumor cell apoptosis [47,48,49], as our electron micrographs show. Furthermore, in the presence of nab-paclitaxel, cell death was additionally managed by mitotic inhibition, specifically, the prevention of microtubule depolymerization, a process essential for cell division [50,51,52]. The surviving tumor cells showed a comparable CD31 expression, cell proliferation activity (Ki67-positive cells in tumors), and reduced the functional integrity of the vascular compartment (reduced CD31 in tumors) but showed a high degree of cellular stress (increased pERK expression, all compared to non-treated controls) in the absence of detectable hypoxic conditions. No new vessel formation was detectable (same small vessel count compared to non-treated controls). This means that the observed ERK activation is related to the attempt of the tumor cells to eliminate the therapeutic drug. Namely, the activation of ERK1/2 by gemcitabine has been linked to drug resistance in pancreatic cancer [40,41] and not to new vessel formation. This means that there was no visible pro-angiogenic contribution in the orthotopic pancreatic tumors in mice as a result of the gemcitabine/paclitaxel chemotherapy.

It is important to note that while the majority of the evidence suggests that hypoxia is a major driver of tumor angiogenesis, here we provide evidence that hypoxia is not required to trigger neo-angiogenesis in orthotopic PANC-1 tumors treated with magnetic hyperthermia and gemcitabine/paclitaxel chemotherapy. In particular, the expression of neuropilin-1, VEGF, and VEGFR2 in tumor cells remained unchanged (unchanged auto- and paracrine VEGF tumor cell signaling), independently of the therapeutic modality (at least under the conditions applied in this study). This means that the mentioned therapies did not impact the known key regulators of tumor neo-angiogenesis. In agreement with this, previous in vitro investigations show that intracellular VEGF expression in PANC-1 cells is unaffected by moderately elevated temperatures (43 °C) but can be affected by higher temperatures (47 °C) [42]. Since autocrine and paracrine VEGF signaling in tumor cells (i.e., the expression of neuropilin-1, VEGF, and VEGFR2) has been linked to the regulation of TGFβ1-stimulated endothelial-mesenchymal transition and tumor fibrosis in PDAC tumors [53], our results provide evidence that the therapeutic modalities may inhibit tumor fibrosis in PDAC tumors. Additionally, our data on tumor oxygen and systemic hemoglobin/red blood cell content show that there were no signs of therapeutically mediated severe hypoxia detectable in the tumors.

It may also be noted that the presence of MNPs in the tumors, which is required for magnetic hyperthermia, may induce the formation of intracellular reactive oxygen species (ROS) as a consequence of their metabolization and degradation. Therefore, it is conceivable that tumor cell death may have occurred, at least in part, through a mechanism called ferroptosis [14]. This is a form of regulated cell death characterized by the accumulation of lipid peroxides and iron-dependent ROS. Nevertheless, the impact of ferroptosis on neo-angiogenesis should be rather low in this specific case, as ferroptosis is known to be associated with the production of pro-angiogenic factors like VEGF as well as the stabilization of HIF-1α, both of which were shown to play a lesser role in the present study (see above).

For clinical translation of the combined therapy method, the most convenient route of MNP application to pancreatic tumors for magnetic hyperthermia is the intratumoral one. In contrast, upon intravenous application, the amount of MNP that reaches the pancreatic tumors will be very low [30]. In our study, we were able to perform two separate hyperthermia sessions with one week in between, with no significant drop-off in thermal dosing in the two sessions. As such, we suggest that the MNPs remained in the orthotopic PANC-1 tumors for least for 7 days. Additionally, a potential MNP leakage from the tumor to other organs should be low as well, since no unintended heating damage was found in organs surrounding the pancreas on experimental day 30.

Taken together, our data show the combination of magnetic hyperthermia with chemotherapy is able to trigger tumor angiogenesis in orthotopic PANC-1 tumors as aresult of damaged and/or stressed tumor cells. In the long term and under certain circumstances, magnetic hyperthermia could be specifically applied to transiently modulate tumor angiogenesis and to improve the accessibility of drugs during oncologic therapies. It could therefore serve as a tool to prevent severe desmoplasia and reduce PDAC resistance to oncological treatments. As such, it might be critical to shift the focus of future research towards investigating the likelihood of hyperthermia having a pro-angiogenic effect on the PDAC tumor microenvironment. Further investigations should unveil the varied responses of tumor cells and their associated stroma cells to hyperthermia and how this could be exploited to regulate the balance of angiogenesis in cancer cells.

## 5. Conclusions

We showed that magnetic hyperthermia in combination with gemcitabine/paclitaxel chemotherapy is able to trigger neo-angiogenesis in orthotopic pancreatic tumors in mice, presumably by mechanisms not directly associated with autocrine and paracrine VEGF signaling. We conclude that such mechanisms are rather induced by therapeutic cell damage and cell stress in tumors that activate specific pro-angiogenic mechanisms, which differ from those seen in hypoxic conditions where HIF1α is typically involved. We attribute the therapeutically induced neo-angiogenesis to the activation of cell stress pathways, like the MAPK pathway. Further explanations are that damaged/stressed tumor cells release specific pro-angiogenic factors other than VEGF or they induce pro-inflammatory responses via the release of inflammatory cytokines. The major contributor to the neo-angiogenic process during the bimodal therapy seems to be the magnetic hyperthermia modality. Since the interplay between various biological factors and pathways is complex in relation to the mechanisms underlying angiogenesis in pancreatic tumors, further studies are needed. These should include the tumor microenvironment and the presence of other genetic alterations within the involved tumor cells. In the long term, the bimodal modality of magnetic hyperthermia and gemcitabine/paclitaxel chemotherapy of pancreatic tumors could serve as a useful tool to prevent severe desmoplasia and reduce tumor resistance during oncological treatments.

## Figures and Tables

**Table 1:** Name and treatment conditions of the animal groups. Animals were randomly assigned into five therapy groups, namely magnetic hyperthermia plus systemic chemotherapy (MHsC), magnetic hyperthermia alone (MH), systemic chemotherapy alone (sC), nanoparticles alone (M), and non-treated animals (N). The treatment given to each animal group is listed in the table. ID: animal group identification.

ID	Nano-Particles	Magnetic Field Exposure	Systemic Chemotherapy	Animal Number
MHsC	Yes	2 cycles	7 cycles	5
MH	Yes	2 cycles	None	5
sC	None	None	7 cycles	5
M	Yes	None	None	5
N	None	None	None	5

**Table 2:** Recorded values of the maximum temperature, *T*_max_, and mean amplitude of the temperature oscillation during the pulsed part of the heating sequence, Δ*T*_osc_, for orthotropic PANC-1 mice during magnetic hyperthermia therapy (for hyperthermic cycles on day 1 and day 7; see Figure 1 and Table 1). The distance between the surface thermal probe and the tumor edge was ca. 1.0 mm in all cases. No: mouse identification; Tumor vol.: volume of tumor on day 0; Injected vol.: injected volume of nanoparticle suspension (1 mg per 100 mm^3^ of tumor) on day 0; MHsC: magnetic hyperthermia + chemotherapy; MH: magnetic hyperthermia.

Animal Group	No.	Tumor Vol. (mm^3^)	Injected Vol. (μL)	*T*_max_ (°C) Day 1	Δ*T*_osc_ (°C) Day 1 Mean ± SD	*T*_max_ (°C) Day 7	Δ*T*_osc_ (°C) Day 7 Mean ± SD
	A	270	54	42.6	0.94 ± 0.42	40.2	1.07 ± 0.11
	B	192	30	39.8	0.45 ± 0.05	39.0	0.54 ± 0.10
MHsC	C	283	50	41.0	0.73 ± 0.17	39.2	0.56 ± 0.09
	D	130	21	39.4	0.48 ± 0.09	38.6	0.29 ± 0.13
	E	165	33	40.8	0.78 ± 0.08	40.5	0.73 ± 0.07
	A	141	20	37.2	0.34 ± 0.15	40.0	0.35 ± 0.12
	B	312	45	37.0	0.38 ± 0.16	40.9	0.29 ± 0.14
MH	C	93	18	38.4	0.35 ± 0.11	40.4	0.36 ± 0.08
	D	157	30	38.2	0.60 ± 0.14	38.8	0.44 ± 0.08
	E	180	36	40.1	0.60 ± 0.18	40.3	0.64 ± 0.05

## Data Availability

Data will be available upon request.

## Supplementary Materials

The following supporting information can be downloaded at https://www.mdpi.com/article/10.3390/cancers16010033/s1, Figure S1: Selected physiological key points of mice bearing orthotopic pancreatic tumors, which were subjected to magnetic hyperthermia. Figure S2: Correlation between measured and modelled Δ*T*_osc_ metrics from the magnetic hyperthermia temperature–time data. Figure S3: Comparable levels of red blood cells and hemoglobin across therapy groups. Figure S4: Stable oxygen content (SO_2_) within the pancreatic tumors across all therapy lines. Figure S5: Correlation of tumor oxygen saturation (SO_2_) and tumor total blood volume in the magnetic hyperthermia-treated tumors at experimental day 29.
