# Supplementary material for: Local Magnetic Hyperthermia and Systemic Gemcitabine/Paclitaxel Chemotherapy Triggers Neo-Angiogenesis in Orthotopic Pancreatic Tumors without Involvement of Auto/Paracrine Tumor Cell VEGF Signaling and Hypoxia
Source: Cancers (Basel). 2023 Dec 20;16(1):33. doi: 10.3390/cancers16010033 (PMC10778317; doi:10.3390/cancers16010033)
Supplement: Supplementary file 1 [file cancers-16-00033-s001.zip › cancers-2634725-supplementary.pdf]

cancers

Supplementary Materials

Local Magnetic Hyperthermia and Systemic Gemcitabine/Paclitaxel Chemotherapy Triggers Neo-angiogenesis in Orthotopic Pancreatic Tumors without Involvement of Auto/Paracrine Tumor Cell VEGF Signaling and Hypoxia

Wisdom O. Maduabuchi [1], Felista L. Tansi [1], Bernd Faenger [1], Paul Southern [2,3], Quentin A. Pankhurst [2,3], Frank Steiniger [4], Martin Westermann [4] and Ingrid Hilger [1,*]

[1] Department of Experimental Radiology, Institute of Diagnostic and Interventional Radiology, Jena University Hospital, Friedrich Schiller University Jena, Am Klinikum 1, 07747 Jena, Germany; felista.tansi@med.uni-jena.de (F.L.T.); bernd.faenger@med.uni-jena.de (B.F.)
[2] Resonant Circuits Limited, 21 Albemarle Street, London W1S 4BS, UK; psouthern@resonantcircuits.com (P.S.); q.pankhurst@ucl.ac.uk (Q.A.P.)
[3] Healthcare Biomagnetics Laboratory, University College London, 21 Albemarle St., London W1S 4BS, UK
[4] Center for Electron Microscopy, Jena University Hospital, Friedrich Schiller University Jena, Ziegelmuehlenweg 1, 07743 Jena, Germany; frank.steiniger@med.uni-jena.de (F.S.); martin.westermann@med.uni-jena.de (M.W.)
* Correspondence: ingrid.hilger@med.uni-jena.de (I.H.); Tel.: +49-3641-9325921

Supplementary Figure S1 shows a representative temperature curve of animals treated with magnetic hyperthermia and representative small animal near infrared fluorescence and micro-CT micrograph showing localization of implanted fluorescent PANC-1 cells.

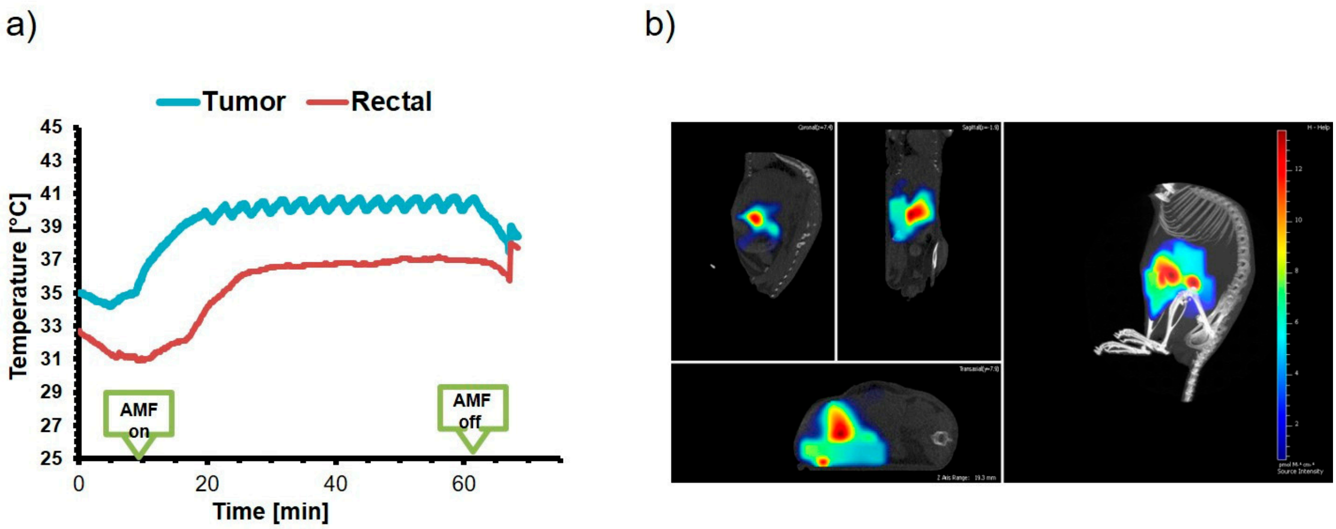

Figure S1: Selected physiological key points of mice bearing orthotopic pancreatic tumors, which were subjected to magnetic hyperthermia. **a)** Representative temperature curve of tumor and rectum in animals treated with magnetic hyperthermia. We used the pulse sequencing of the magnetic field to reduce the damaging flow of heat away from the tumor according to Tansi et al. 2021*. **b)** Representative micro-CT image of fluorescent PANC-1 cells implanted orthotopically (mKate2: excitation at 445 nm and emission at 598 nm, in vivo imaging system IVIS-Spectrum CT from Perkin Elmer, f-stop: 2 and binning: small) showing the coronal, sagittal and transaxial views of PANC-1 cells localization within the mouse directly before treatment with magnetic hyperthermia. Fluorescence intensities were depicted as average radiance efficiency (p/s/cm2/sr]/[µW/cm^2) showing false color coded fluorescence images.

* Tansi FL, Maduabuchi WO, Hirsch M, Southern P, Hattersley S, Quaas R, Teichgräber U, Pankhurst QA, Hilger I. Deep-tissue localization of magnetic field hyperthermia using pulse sequencing. Int J Hyperthermia. 2021;38(1):743-754. doi: 10.1080/02656736.2021.1912412. PMID: 33941016.

Supplementary Figure S2 shows the correlation between measured and modelled ΔT_{osc} metrics from the magnetic hyperthermia temperature-time data.

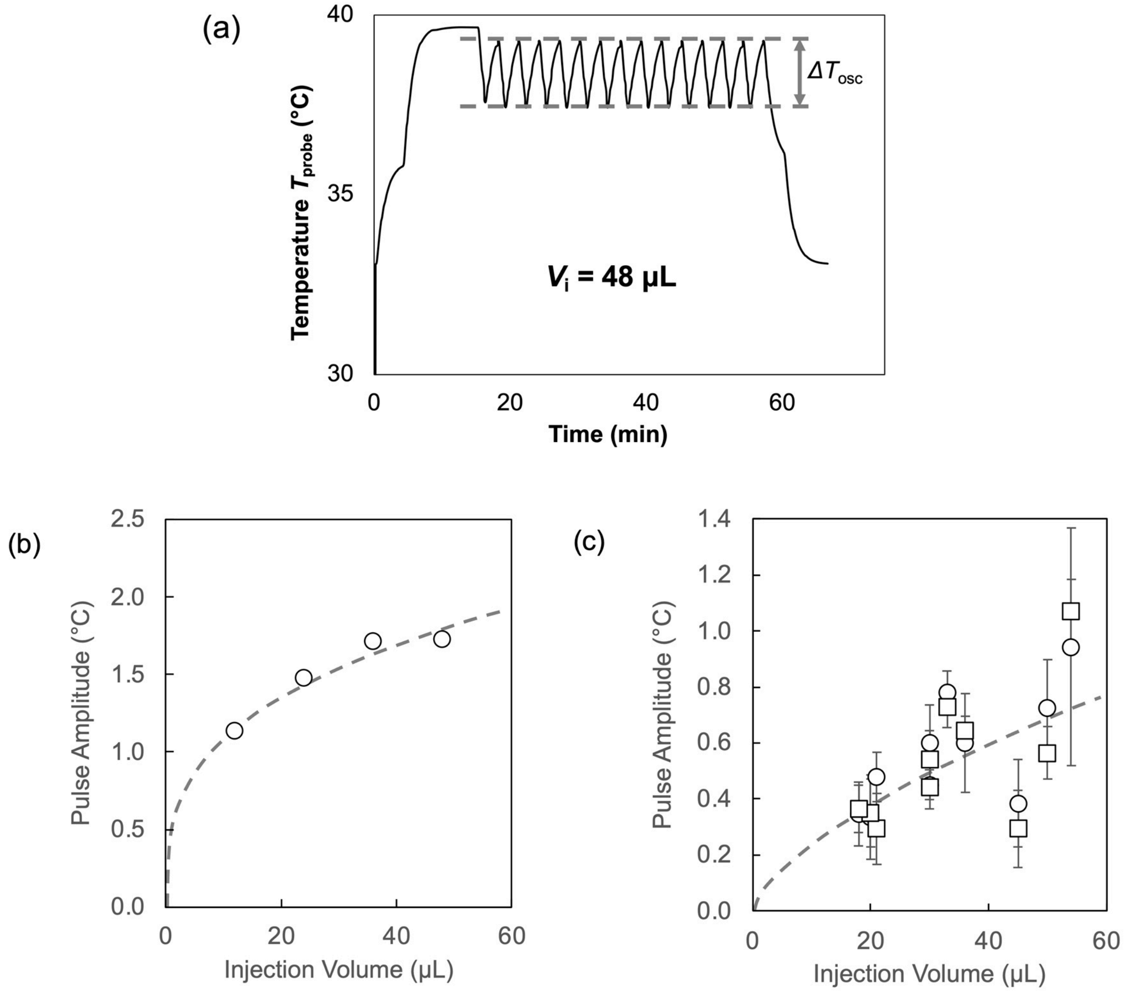

Figure S2: Correlation between measured and modelled ΔT_{osc} metrics from the magnetic hyperthermia temperature-time data. **a)** Modelled temperature-time curve for a thermal probe positioned 1 mm from the edge of an orthotopic pancreatic tumor of volume 240 mm^3 injected with 48 μL of RCL-01 – as described in Tansi et al. * – assuming that the rate of heat deposition into the tissue oscillated between 1.3 and 3.0 W/cm^3 throughout the pulsed heating sequence. **b)** Modelled variation in the "pulse amplitude", ΔT_{osc}, as a function of the injection volume, for thermal probes positioned at 1 mm from the tumor edge, for four different tumor/injection volumes, wherein $V_{injection}$ = 0.2 V_{tumor}. The dashed line is a guide to the eye only. **c)** Measured variation in ΔT_{osc} for the MH and MHsC groups of animals as recorded on day 1 (squares) and on day 7 (circles). The dashed line is a guide to the eye only but follows the same functional dependence as that used for the modelled data. The measured ΔT_{osc} values are ca. 1/3 the magnitude of the modelled values, indicating that the rate of heat transfer in the experimental groups was at most ca. 1.0 W/cm^3.

* Tansi FL, Maduabuchi WO, Hirsch M, Southern P, Hattersley S, Quaas R, Teichgräber U, Pankhurst QA, Hilger I. Deep-tissue localization of magnetic field hyperthermia using pulse sequencing. Int J Hyperthermia. 2021;38(1):743-754. doi: 10.1080/02656736.2021.1912412. PMID: 33941016.

Supplementary Figure S3 shows the level of red blood cells and hemoglobin across therapy groups. Our analysis reveals comparable level of red blood cells and hemoglobin in all groups at the end of therapy (d30). Additionally, during the course of treatment animals of all groups showed comparable total hemoglobin in the tumor (MSOT imaging).

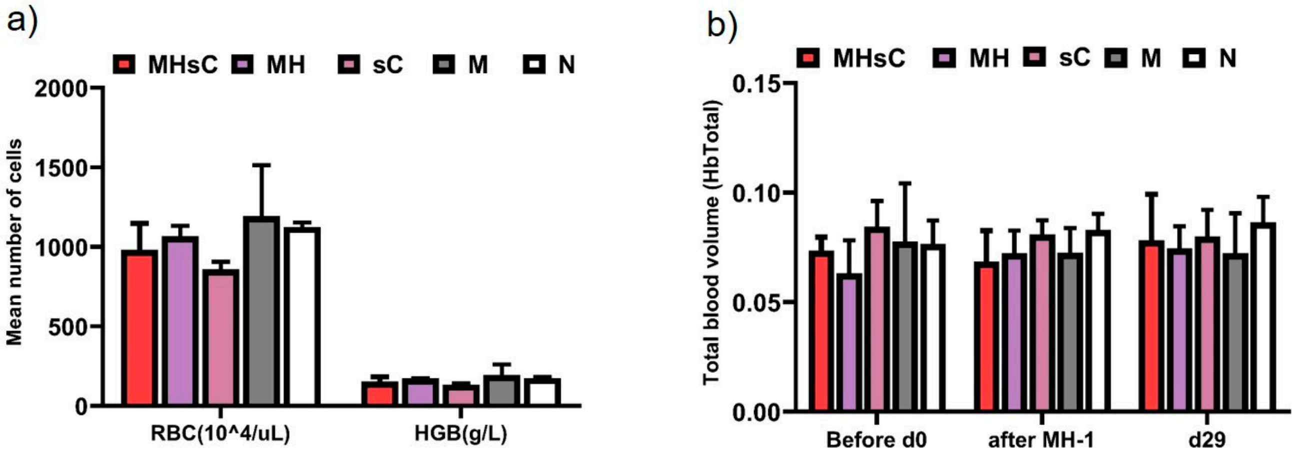

Figure S3: Comparable levels of red blood cells and hemoglobin across therapy group. a) There was no significant effect of the bimodality therapy, hyperthermia alone or chemotherapy alone on the red blood cells count or hemoglobin level by the end of therapy (day 30). b) Tumor total blood volume (total hemoglobin) measured via MSOT two days before MNP application (day 0), 2 days after first hyperthermia and at the end of therapy (day 29). Each bar represents mean ± SEM (n = 4 mice/group for MHsC while n = 5 mice/group for other groups). Animal groups: MHsC = magnetic hyperthermia + chemotherapy, MH = magnetic hyperthermia, sC = systemic chemotherapy, M = MNP alone, N = non-treated.

Supplementary Figure S4 shows that tumor oxygen saturation was not significantly altered across all treatment groups during the course of the investigation.

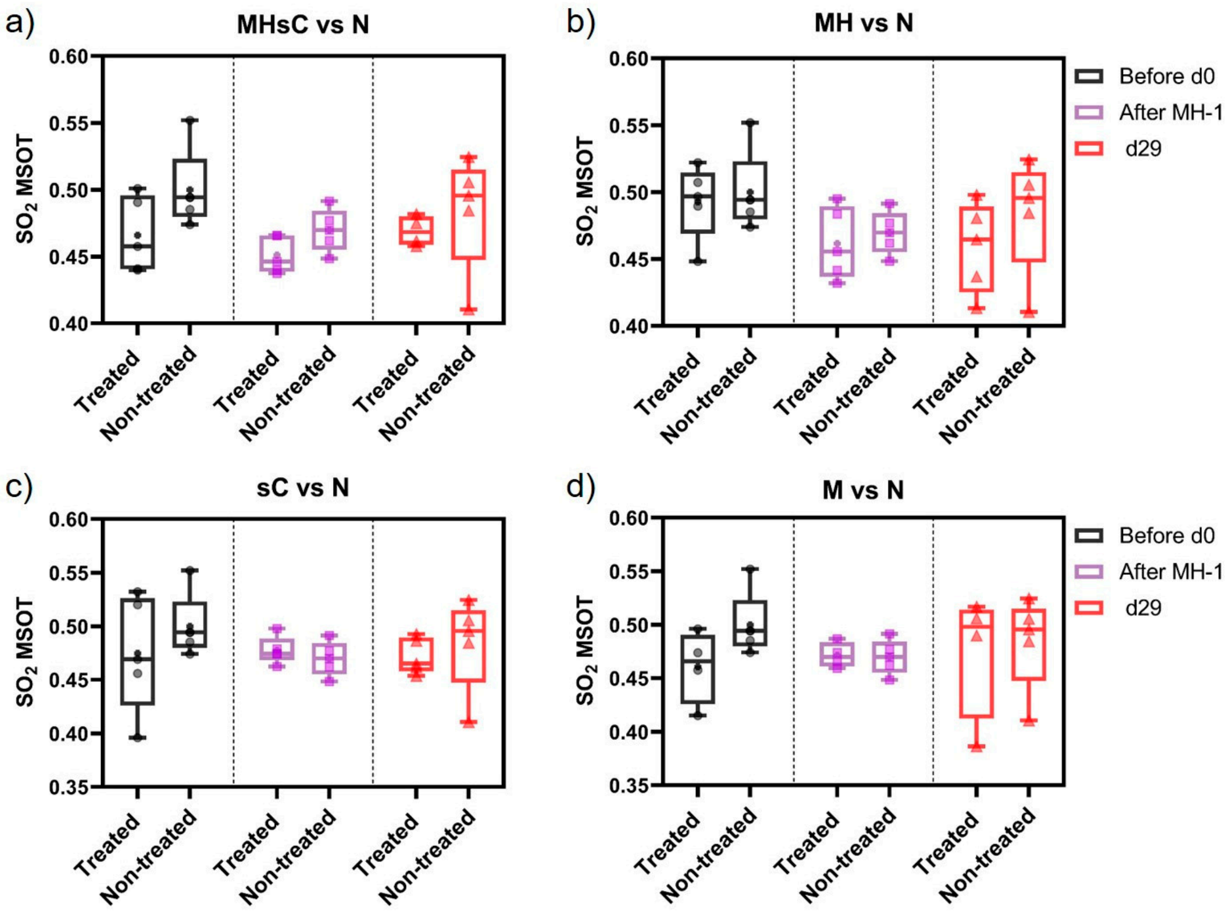

Figure S4: Stable oxygen content (SO2) within the pancreatic tumors across all therapy line. Oxygen saturation was measured via MSOT two days before MNP application (d0), two days after first hyperthermia (MH-1) and at the end of therapy (d29). a) SO2 in the tumor of animals treated with the bimodality therapy (MH + chemotherapy) versus non-treated (control). b) Comparable SO2 level with MH treatment compared to non-treated animal. c) SO2 level with chemotherapy. d) Comparable levels of SO2 in MNP-treated tumors by the end of therapy compared to the non-treated. (n = 5 mice/group except d29 MHsC is n = 4). Animal groups: MHsC = magnetic hyperthermia + chemotherapy, MH = magnetic hyperthermia, sC = systemic chemotherapy, M = MNP alone, N = non-treated.

Supplementary Figure S5 shows the correlation of tumor oxygen saturation (SO_2) and tumor total blood volume in the magnetic hyperthermia-treated tumors at experimental day 29.

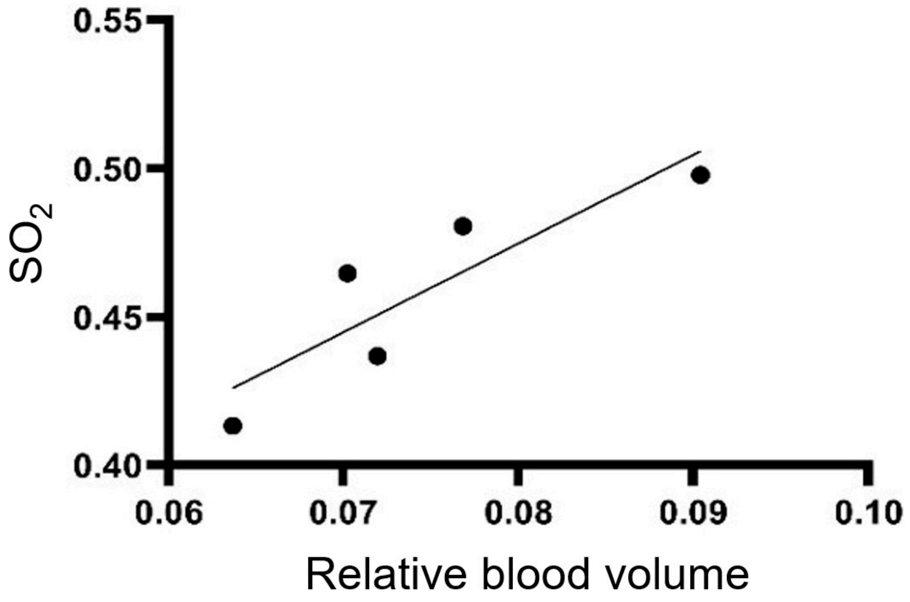

Figure S5: Correlation of tumor oxygen saturation (SO_2,) and tumor total blood volume in the magnetic hyperthermia-treated residual tumors at experimental day 29. Tumor relative blood volume (RBV, see Methods) and tumor oxygen saturation (see Methods) were correlated using Pearson's formula with $r = 0.88$, $p = 0.047$, $n = 5$ mice/group.
